# MXene- and MOF-Based Hydrogels: Emerging Platforms for Electrochemical Biosensing and Health Monitoring

**DOI:** 10.3390/mi17020267

**Published:** 2026-02-20

**Authors:** Kandaswamy Theyagarajan, Sairaman Saikrithika, Young-Joon Kim

**Affiliations:** 1 Department of Electronic Engineering, Gachon University, Seongnam 13120, Gyeonggi-Do, Republic of Korea; 2 Department of Semiconductor Engineering, Gachon University, Seongnam 13120, Gyeonggi-Do, Republic of Korea

**Keywords:** electrochemical sensor, modified electrode, wearable sensor, biosensor, soft materials

## Abstract

Smart healthcare is rapidly emerging as a transformative paradigm, enabling simultaneous health monitoring, therapeutic intervention, and early prediction of disease onset. In this context, electrochemical monitoring systems have attracted growing interest due to their cost-effectiveness, ease of operation, miniaturization and compatibility with wearable platforms. Accordingly, conductive hydrogel-based electrochemical (bio)sensors have gained significant attention for health monitoring owing to their soft mechanical properties, high water content, excellent biocompatibility, and ability to form intimate, conformal interfaces with biological tissues. Their three-dimensional polymeric networks facilitate efficient ion transport and mechanical flexibility, making them particularly suitable for wearable and noninvasive sensing and monitoring applications. However, the intrinsically limited conductivity and catalytic activity of pristine hydrogels often constrain their electrochemical performance. To overcome these limitations, functional nanomaterials such as metal–organic frameworks (MOFs) and MXene (MX) nanosheets have been increasingly integrated into hydrogel matrices to enhance conductivity and electrochemical activity. This review provides a comprehensive and critical comparison of recent advances in MOF- and MX-integrated conductive hydrogels for electrochemical health monitoring. In addition to material design strategies and sensing performance, emerging trends in data-driven sensing aimed at improving signal interpretation and multi-analyte discrimination are systematically discussed. Key challenges related to long-term stability, biocompatibility, scalability, and intelligent system integration are critically assessed, and the future potential of these platforms within closed-loop architectures is highlighted, paving the way for next-generation conductive hydrogel-based electrochemical sensors in smart healthcare applications.

## 1. Introduction

The rapid expansion of personalized healthcare, preventive medicine, and point-of-care diagnostics towards smart healthcare has significantly increased the demand for continuous, real-time, and noninvasive health monitoring technologies [1,2]. Conventional diagnostic approaches, including centralized laboratory testing and imaging-based techniques, often rely on complex instrumentation, trained personnel, and invasive sampling procedures, which limit their suitability for continuous monitoring [3,4]. These limitations are particularly problematic for chronic disease management, early-stage diagnosis, and long-term physiological tracking, where access to dynamic biochemical information is essential. Consequently, there is a growing need for miniaturized, portable, wearable, and user-friendly sensing systems capable of operating directly at the human-device interface [5,6]. In this context, electrochemical sensors have emerged as one of the most promising analytical platforms for health monitoring owing to their intrinsic advantages, including high sensitivity, rapid response, low power consumption, and compatibility with microfabrication and wearable technologies [7,8,9]. In addition, electrochemical sensors are versatile, cost-effective, easy to operate, and amenable to large-scale fabrication [10,11]. Their ability to transduce biochemical interactions into electrical signals enables quantitative analysis of a wide range of biomarkers, including metabolites, electrolytes, proteins, drugs, and antioxidants, in diverse biological fluids [12,13]. However, the performance of electrochemical sensors is strongly influenced by the physical and chemical properties of the sensing interface, particularly when intimate and stable contact with soft, dynamic biological tissues is required [14].

In this context, materials that combine mechanical compliance with electrochemical functionality have become central to the design of next-generation sensors. Among these, conductive hydrogel-based sensing and monitoring systems have attracted considerable attention due to their tissue-like softness, high water content, and excellent biocompatibility [15,16]. Hydrogels represent a unique class of materials in which three-dimensional polymeric networks enable efficient ion transport while supporting electronic conductivity through the incorporation of conductive fillers or redox-active species [17,18]. Their porous and hydrated structures facilitate effective diffusion of analytes and electrolytes, making them particularly well suited for electrochemical sensing in biofluids such as sweat, tears, saliva, and interstitial fluid [19]. Moreover, the intrinsic flexibility and elasticity of hydrogels allow stable signal acquisition even under mechanical deformation, a critical requirement for wearable health-monitoring devices [20]. Despite these advantages, pristine hydrogels often suffer from inherent limitations that restrict their electrochemical performance. These include relatively low electrical conductivity compared to inorganic conductors, limited electrocatalytic activity toward specific analytes, and insufficient selectivity in complex biological environments [21,22]. In addition, the absence of well-defined and abundant active sites within hydrogel networks can impede efficient signal amplification and reduce sensitivity, particularly for low-abundance biomarkers [23]. Consequently, substantial research efforts have focused on enhancing the physicochemical and electrical properties of hydrogels through the incorporation of functional nanomaterials. In this regard, metal–organic frameworks (MOFs) have emerged as particularly attractive candidates for integration into hydrogel matrices owing to their highly tunable porous architecture, large specific surface areas, and versatile coordination chemistry [24]. Composed of metal ions or clusters coordinated with organic linkers, MOFs allow precise control over pore size, surface functionality, and chemical affinity. These characteristics render MOFs highly effective for analyte adsorption, molecular recognition, and electrocatalytic processes [25]. In electrochemical sensing applications, MOFs can introduce abundant active sites and promote analyte preconcentration at the electrode interface, thereby significantly enhancing sensitivity and selectivity [26]. In Parallel, two-dimensional MXenes (MX) have gained increasing attention as advanced conductive materials for electrochemical applications. Typically derived from layered MAX phases, MX exhibits exceptional electrical conductivity, high hydrophilicity, and rich surface chemistry with functional terminations such as -OH, -O, and -F [27,28]. These features facilitate strong interfacial interactions with polymers and biomolecules, as well as rapid charge transfer during electrochemical reactions. When incorporated into hydrogels, MXs can form interconnected conductive networks that markedly improve electron transport while preserving the mechanical flexibility of the hydrogel matrix [29]. Collectively, the synergistic integration of MOFs and MXs into hydrogels has emerged as a powerful strategy to overcome the intrinsic limitations of pristine hydrogels, enabling high-performance electrochemical platforms for smart healthcare applications.

Accordingly, the field of conductive hydrogels for electrochemical sensing and monitoring applications has expanded rapidly, reflecting both its novelty and growing scientific interest. Bibliometric analyses based on searches in Scopus and the Web of Science reveal a steady increase in research publications focused on hydrogel-based electrochemical analysis, underscoring the accelerating research efforts devoted to the design and development of advanced hydrogel-enabled monitoring systems. These trends highlight the timeliness and significance of critically reviewing this emerging area. In this review, recent progress in conductive hydrogel-based electrochemical sensors for health monitoring is comprehensively examined, with particular emphasis on the roles of MX and MOFs as functional additives. The review is structured to first introduce the fundamental properties of conductive hydrogels, followed by detailed discussions of MOF-integrated and MX-integrated hydrogel systems. Hybrid MX-MOF hydrogel architectures are then highlighted to illustrate synergistic material effects. Finally, emerging trends toward intelligent, data-driven, and closed-loop sensing systems are discussed, along with current challenges and future perspectives. Ultimately, this review aims to provide a coherent framework for the rational design of next-generation conductive hydrogel-based electrochemical sensing systems for smart and intelligent health monitoring.

## 2. Conductive Hydrogels: Fundamentals and Properties

Conductive hydrogels are soft materials composed of crosslinked polymer networks that combine high water content with the ability to transport electrical charges. Unlike conventional solid conductors, these materials operate through ionic conduction, electronic conduction, or mixed ion-electron transport mechanisms, making them particularly suitable for electrochemical sensing in aqueous and biological environments [30]. Their three-dimensional porous architecture enables efficient diffusion of electrolytes and analytes while maintaining mechanical compliance, which is essential for stable operation under deformation [31]. At the molecular level, conductive hydrogels consist of hydrophilic polymer chains interconnected through physical or chemical crosslinking. Common polymer backbones include polyacrylamide, polyvinyl alcohol, alginate, chitosan, gelatin, and other synthetic or natural polymers [32,33]. The presence of abundant hydrophilic functional groups enables extensive water uptake, forming interconnected channels that facilitate mass transport. The degree of crosslinking plays a critical role in determining pore size, swelling behavior, and mechanical strength, thereby influencing both electrochemical performance and structural stability [34].

The electrical conductivity of hydrogels can be further enhanced through various strategies, including the incorporation of ionic species, redox-active components, conductive polymers, or conductive nanomaterials [35]. Ionic conductive hydrogels primarily rely on ion migration within the hydrated network, whereas electronically conductive hydrogels enable electron transport through percolated conductive pathways [36]. In many electrochemical sensing applications, mixed-conduction hydrogels are preferred, as they support both faradaic electrochemical reactions and efficient ion exchange at the electrode-electrolyte interface. The electrochemical behavior of conductive hydrogels is governed by the interplay between ion mobility, electron transfer kinetics, and interfacial charge distribution [37]. The hydrated polymer matrix provides a low-resistance pathway for ionic diffusion, minimizing concentration polarization during electrochemical measurements, while electronically conductive components enhance charge transfer efficiency, reducing internal resistance and improving signal stability. Importantly, the soft and porous nature of hydrogels promotes intimate contact between the sensing interface and the surrounding electrolyte or biological environment, facilitating rapid equilibration and minimizing signal drift caused by interfacial delamination [38]. However, excessive swelling or dehydration can adversely affect conductivity and mechanical integrity, highlighting the need for careful material design and environmental control.

One of the defining advantages of conductive hydrogels is their mechanical adaptability. Their elastic and viscoelastic properties can be tailored to closely match those of biological tissues, enabling conformal contact with irregular and dynamic surfaces [39]. Properties such as stretchability, compressibility and self-recovery are particularly valuable for wearable and skin-mounted electrochemical sensing applications, where repeated mechanical deformation is unavoidable. Mechanical deformation can influence electrochemical performance by altering conductive pathways and pore structures; thus, well-designed hydrogels maintain continuous charge-transport networks even under strain to ensure consistent signal output [40]. In contrast, poorly integrated conductive components may suffer from cracking or phase separation, leading to signal instability. Achieving mechanical robustness without compromising electrochemical functionality, therefore, remains a critical design challenge. Hydrogels inherently exhibit favorable biocompatibility due to their high water content and soft texture, which reduces irritation and mechanical mismatch at biointerfaces. This property is particularly important for sensors intended for prolonged contact with skin or biological fluids [41]. In addition, the polymer can serve as a reservoir for biomolecules or recognition elements, enabling localized interactions while protecting sensitive components from harsh external conditions. From an electrochemical perspective, the hydrogel interface plays a crucial role in modulating analyte accessibility and signal transduction. The hydrated network supports efficient diffusion of target molecules while limiting non-specific adsorption, thereby improving selectivity. Furthermore, the ability to engineer surface chemistry within hydrogels allows precise control over wettability, charge distribution, and interfacial kinetics [42].

**Figure 1:**
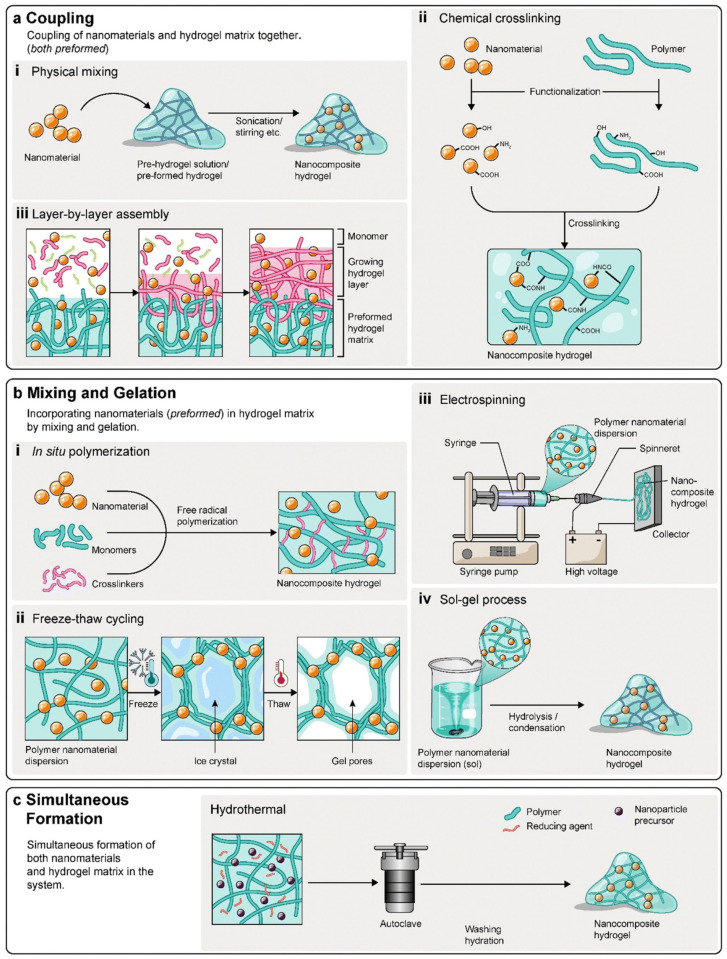
Schematic illustration of different strategies for integration of nanomaterials into hydrogel matrices: (**a**) coupling, (**b**) mixing and gelation and (**c**) simultaneous formation. Reproduced with permission from [18].

Despite these advantages, pristine conductive hydrogels often exhibit limitations that restrict their performance in demanding electrochemical applications. Their intrinsic electrical conductivity is generally lower than that of inorganic conductors, which can limit sensitivity and signal-to-noise ratios. Moreover, the lack of well-defined catalytic or recognition sites reduces selectivity toward specific analytes, particularly in complex biological matrices. Long-term stability also remains a challenge, as repeated swelling-deswelling cycles, mechanical fatigue, and environmental fluctuations can alter network structure and conductivity [43,44,45]. These constraints have motivated extensive research efforts focused on material hybridization strategies, in which functional nanomaterials are incorporated to enhance charge transport, introduce catalytic activity, and stabilize electrochemical responses. Figure 1 portrays different strategies commonly followed for nanomaterial integration in hydrogel matrices. In the following sections, the integration of advanced nanomaterials, specifically MOFs and MXs, into conductive hydrogels is discussed in detail, highlighting how these hybrid systems overcome the intrinsic limitations of hydrogel-based electrochemical sensors and enable high-performance platforms for health monitoring.

## 3. Metal–Organic Framework-Integrated Hydrogels

**Figure 2:**
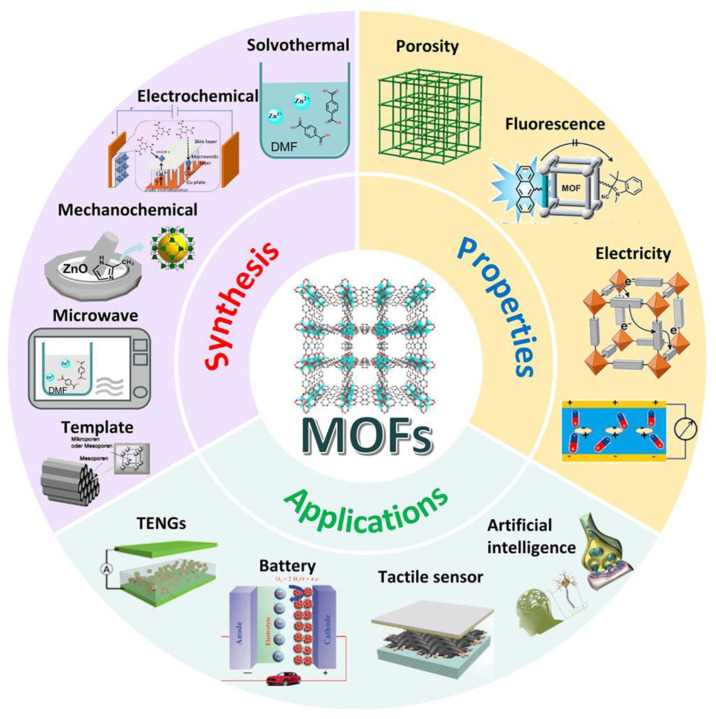
Graphical representation of MOF synthesis, key properties, and representative applications. Reproduced with permission from [58].

Metal–organic frameworks (MOFs) are crystalline porous materials composed of metal ions or metal clusters coordinated with organic ligands, forming well-defined three-dimensional architectures [46]. Their modular construction enables precise control over pore size, surface chemistry, and framework topology, rendering MOFs highly attractive for electrochemical sensing applications (Figure 2) [47]. When incorporated into conductive hydrogels, MOFs introduce structural and chemical functionalities that are difficult to achieve with polymer networks alone. A defining characteristic of MOFs is their exceptionally high specific surface area combined with tunable porosity [48]. By judicious selection of metal nodes and organic linkers, MOFs can be engineered with micro- or mesoporous structures tailored to the size, charge, and chemical nature of target analytes (Figure 2). This structural tunability allows MOFs to function as molecular sieves or adsorption scaffolds within hydrogel matrices, thereby enhancing analyte preconcentration at the sensing interface [49]. Within conductive hydrogels, the porous MOF framework facilitates electrolyte penetration and analyte diffusion while preserving the hydrated environment required for electrochemical reactions. Moreover, functional groups present on organic linkers enable specific chemical interactions, such as hydrogen bonding, coordination, or π-π stacking, which can significantly improve selectivity toward particular biomolecules or metabolites [50,51]. Collectively, these attributes make MOFs effective platforms for integrating molecular recognition and transport functions into soft sensing materials.

Beyond their structural advantages, MOFs can actively contribute to electrochemical signal generation through their metal centers. Redox-active metals such as Cu, Ni, Co, Fe, and Mn can undergo redox reactions, imparting intrinsic electrochemical activity [52]. Within conductive hydrogel matrices, these metal centers serve as catalytic sites that lower reaction overpotentials and enhance faradaic responses toward specific analytes. MOFs can further promote electrocatalytic processes by stabilizing reaction intermediates or facilitating electron transfer through metal–ligand coordination pathways [53]. Although many pristine MOFs exhibit limited electrical conductivity, their catalytic contributions are often amplified when coupled with conductive polymer networks or secondary conductive fillers. In such hybrid systems, MOFs function as chemically active domains, while the hydrogel matrix supports efficient ion transport and mechanical compliance [54]. The incorporation of MOFs into conductive hydrogels can be achieved through various strategies, including physical embedding, in situ growth within the polymer network, or post-synthetic modification. Simple physical dispersion enables facile fabrication but may suffer from particle aggregation or leaching if interfacial interactions are insufficient. In contrast, in situ growth of MOFs within hydrogels typically yields stronger interfacial adhesion and more uniform distribution, leading to improved mechanical integrity and electrochemical stability [55,56].

From a materials design perspective, the hydrogel matrix serves as a soft scaffold that buffers the intrinsic rigidity and brittleness of MOFs, enabling deformation-tolerant sensing interfaces. Simultaneously, MOFs reinforce the hydrogel network by introducing rigid, porous domains that can enhance structural robustness and long-term stability. Achieving an optimal balance among MOF loading, hydrogel crosslinking density, and interfacial compatibility is therefore essential for maximizing electrochemical performance. MOF-integrated conductive hydrogels synergistically combine mechanical softness with high chemical functionality, enabling enhanced analyte adsorption, catalytic activity, and signal modulation. These attributes are particularly valuable for electrochemical health monitoring applications, where high sensitivity and selectivity must be achieved without compromising flexibility or biocompatibility. Despite these advantages, several challenges remain, including the limited intrinsic conductivity of many MOFs, potential framework instability in aqueous or biological environments, and metal ion leaching during prolonged operation [57]. Addressing these issues requires careful materials engineering, such as the selection of chemically robust MOFs, optimization of framework-polymer interactions, and incorporation of complementary conductive components. These considerations have driven the development of advanced MOF-based hydrogel architectures, which are discussed in subsequent sections through representative electrochemical sensing studies.

## 4. MXene-Integrated Hydrogels

**Figure 3:**
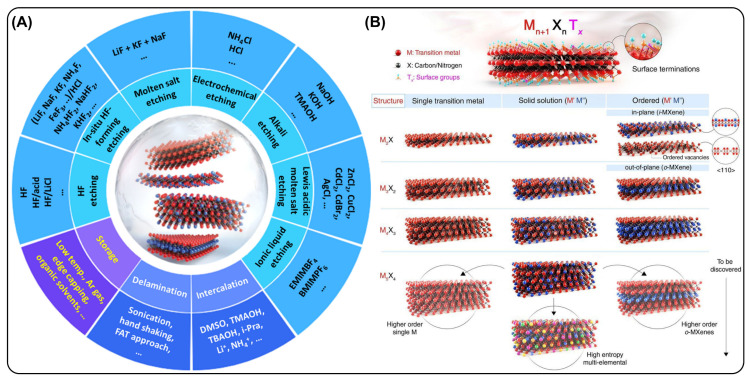
(**A**) Graphical overview of MXene synthesis via different etching methods, delamination strategies and storage conditions. Reproduced with permission from [70]. (**B**) Graphical representation of different MXene families. Reproduced with permission from [71].

MXenes (MXs) are a rapidly expanding family of two-dimensional transition-metal carbides, nitrides, and carbonitrides derived from layered MAX phases through selective etching processes (Figure 3A). Their unique combination of metallic conductivity, hydrophilicity, and rich surface chemistry has positioned MXs as highly effective conductive components for electrochemical sensing platforms [59,60]. When integrated into conductive hydrogels, MXs address key limitations of polymeric networks by establishing efficient electron-transport pathways while preserving mechanical compliance and interfacial adaptability [61]. Structurally, MXs consist of layered transition-metal atoms (e.g., Ti, V, Nb, Mo) interleaved with carbon or nitrogen layers (Figure 3B). During synthesis, surface terminations such as -OH, -O, and -F are introduced, imparting strong hydrophilicity and chemical reactivity. These surface functional groups enable excellent dispersion of MX nanosheets in aqueous environments and promote strong interactions with polymer chains through hydrogen bonding, electrostatic attraction, or coordination interactions [62]. Within hydrogel matrices, the high aspect ratio and planar morphology of MX nanosheets facilitate the formation of interconnected networks. This percolated structure enables continuous charge-transport pathways at relatively low filler loadings, which is advantageous for maintaining hydrogel softness, elasticity and stretchability while achieving high electrical conductivity. A key advantage of MXs lies in their exceptionally high intrinsic electrical conductivity, which approaches or surpasses that of many conventional conductive fillers [63]. In conductive hydrogels, MX functions as an efficient electron highway, significantly reducing internal resistance and enhancing charge-transfer kinetics at the electrode-electrolyte interface. This capability is particularly critical for electrochemical sensing applications that require rapid, sensitive, and reproducible current or impedance responses. Beyond electronic conductivity, MXs’ surface terminations actively contribute to interfacial charge exchange by participating in redox processes or facilitating the adsorption of electroactive species [64]. Consequently, MX-integrated hydrogels often exhibit improved signal stability, faster response times, and enhanced sensitivity compared to hydrogel systems lacking conductive nanofillers.

Interfacial interactions between MX nanosheets and the polymer network strongly govern the performance of MX-based conductive hydrogels. Robust interfacial bonding improves mechanical integrity, suppresses nanosheet restacking, and ensures homogeneous dispersion throughout the hydrogel matrix. These interactions also impart excellent strain tolerance, allowing conductive pathways to remain intact under stretching, bending, or compression [65,66]. From a sensing perspective, the hydrogel environment stabilizes MXs against aggregation while maintaining accessibility to their electrochemically active surfaces. Simultaneously, the hydrated polymer matrix supports efficient ion transport, enabling synergistic ion-electron conduction that is essential for electrochemical signal transduction in biological media [67]. Moreover, MX-integrated conductive hydrogels therefore offer several advantages for electrochemical health monitoring, including high conductivity, mechanical flexibility, and strong interfacial compatibility with polymer matrices. These attributes support stable signal generation under dynamic physiological conditions and enable long-term operation in wearable and biointegrated sensing devices [68,69]. In addition, the versatile surface chemistry of MX allows facile functionalization with recognition elements, enzymes, or catalytic components, further expanding their applicability in selective and multifunctional sensing platforms.

Despite these advantages, several challenges remain. MXs are susceptible to oxidation under ambient and aqueous conditions, which can progressively degrade electrical conductivity and electrochemical performance. Consequently, controlling oxidation, ensuring long-term material stability, and optimizing MX loading without compromising hydrogel mechanics remain critical design considerations. These challenges have stimulated the development of advanced MX-based hydrogel architectures, including hybrid systems that combine MXs with complementary functional materials. Having outlined the fundamental properties and functional roles of conductive hydrogels, MOF-integrated hydrogels, MX-based hydrogels, and their hybrid architectures, the following sections shift toward a critical discussion of representative electrochemical sensing platforms. Recent studies employing these material systems for health monitoring are systematically reviewed, with emphasis on sensing mechanisms, analytical performance, device configuration, and real-sample applicability.

## 5. Electroanalytical Techniques for Hybrid Hydrogel-Based Sensors

Some of the commonly employed electroanalytical techniques for MXene- and MOF-integrated hydrogel-based sensors include differential pulse voltammetry (DPV), amperometry and square wave voltammetry (SWV). Less frequently, techniques such as electrochemical impedance spectroscopy (EIS), linear sweep voltammetry (LSV), and cyclic voltammetry (CV) are also utilized. Owing to its high sensitivity, DPV is one of the most widely used electroanalytical techniques for conductive hybrid hydrogels. It belongs to the family of pulse voltammetric methods, in which a series of small potential pulses is superimposed onto a slowly increasing base potential. The current is measured immediately before each pulse and at the end of the pulse, and the difference in current is plotted against the applied potential. This differential measurement enhances the signal-to-noise ratio, minimizes capacitive (non-faradaic) current, and makes DPV particularly suitable for trace-level analysis in health monitoring applications [9]. SWV can be considered an advanced pulse voltammetric technique related to DPV. In SWV, a symmetrical square-wave potential is superimposed onto a staircase potential ramp. The current is measured at the end of both the forward and reverse pulses, and the difference between these currents is plotted as a function of applied potential. This approach effectively suppresses capacitive contributions and isolates faradaic responses arising from redox reactions. Consequently, SWV offers very high sensitivity, low detection limits, and rapid data acquisition, making it well-suited for fast and trace electrochemical sensing [13].

Amperometry is another commonly employed technique, in which a constant potential is applied to the working electrode and the resulting oxidation or reduction current is recorded as a function of time. Under steady-state conditions, the measured current is proportional to the concentration of the electroactive species [30]. Depending on the sensing configuration and application requirements, amperometry can be performed under static, hydrodynamic, pulsed, or differential pulse modes. It is widely used due to its high sensitivity, rapid response, compatibility with miniaturized and wearable systems, and suitability for continuous real-time monitoring [13]. CV is primarily used to investigate redox behavior, understand electrode processes, and characterize new electrode materials. In CV, the potential is swept linearly in a triangular waveform, first in the forward direction and then reversed, while the resulting current is recorded as a function of applied potential. CV is simple, versatile, and widely applied, providing valuable mechanistic insights and enabling comparison of different electrode modifications. On the other hand, EIS is a non-destructive and highly powerful technique used to study charge-transfer processes, interfacial properties, and mass-transport phenomena at electrode surfaces. In EIS, a small-amplitude AC voltage is applied over a range of frequencies, and the resulting AC response is measured. Rather than driving the system far from equilibrium, EIS perturbs the system slightly and analyzes its frequency-dependent response [21]. Because it is highly sensitive to interfacial changes, EIS is particularly useful for monitoring binding events, evaluating electrode modifications, confirming biomolecule immobilization, and enabling label-free detection.

## 6. MOF-Integrated Hydrogels for Electrochemical Health Monitoring

A flexible, non-enzymatic electrochemical contact-lens sensor was developed by integrating a nickel-cobalt-BDC MOF into a transparent hydrogel matrix for direct urea sensing in tear fluid [72]. Urea is an important metabolic biomarker associated with renal function, hydration status, and metabolic disorders, and its noninvasive monitoring in tears offers a painless alternative to blood-based diagnostics. The system leverages the high porosity and abundant redox-active Ni and Co centers of the MOF to enhance the electrocatalytic oxidation of urea, while the hydrogel matrix provides mechanical flexibility, optical transparency, and biocompatibility suitable for wearable ocular applications. During electrochemical operation, the Ni^2+^ species are initially oxidized to higher valence states, which facilitate the activation of Co centers into catalytically active species that promote the electrooxidation of urea. Moreover, the synergistic interaction between nickel and cobalt species enhances charge transfer and catalytic activity toward urea oxidation. Good selectivity against common electroactive interferents and satisfactory performance in simulated tear samples demonstrate its potential for continuous, wearable tear-based urea monitoring. Another portable electrochemical sensor was developed using the same NiCo-BDC MOF embedded in a chitosan/polyacrylamine hydrogel to enable sensitive detection of adrenaline in biological fluids [73]. Adrenaline is a crucial catecholamine involved in stress response and cardiovascular function, and its accurate quantification is vital for diagnosing cardiac events and monitoring physiological stress. The sensor integrates the high surface area, abundant active sites, and redox activity of the NiCo-BTC MOF with the flexible, conductive hydrogel matrix, enhancing analyte accessibility and electron transfer at the interface. Structural and electrochemical characterization confirmed uniform dispersion of the MOF and strong synergistic interactions between Ni and Co centers. The device exhibited a wide linear detection range and low nanomolar detection limits for adrenaline, with good selectivity against common interferents and reliable performance in real sample analyses, indicating its promise for point-of-care catecholamine monitoring.

**Figure 4:**
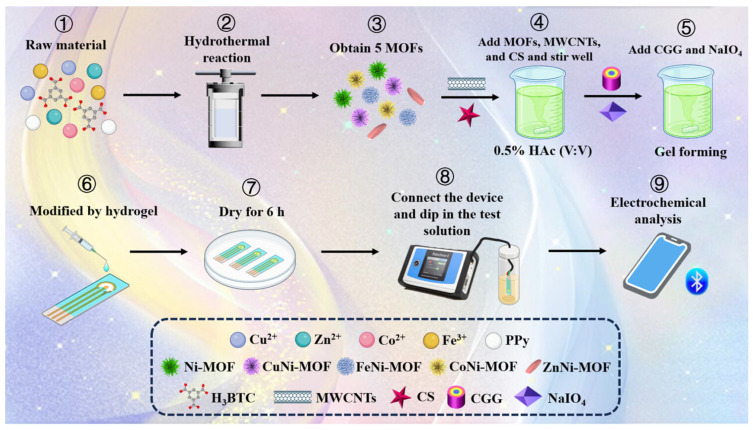
Schematic illustration for the stepwise synthesis and fabrication (1–7) of a MOF-integrated hydrogel sensor, along with its electrochemical activity with smartphone-based display output (8,9). Reproduced with permission from [75].

Similarly, a bimetallic MOF was developed, this time with an iron-cobalt-BDC MOF dispersed on a polyvinyl alcohol/chitosan multi-matrix hydrogel for the electrochemical detection of lactic acid in sweat [74]. Lactic acid is a crucial metabolic biomarker associated with muscle fatigue, exercise intensity, and metabolic disorders, and its real-time monitoring in sweat enables noninvasive assessment of physiological stress and athletic performance. The high surface area and abundant catalytic sites of the Fe-Co-BDC MOF, combined with the conductive, flexible hydrogel network, facilitated efficient electron transfer and analyte accessibility at the electrode interface. Structural characterization confirmed homogeneous MOF dispersion and a porous hydrogel structure that promotes sweat uptake and analyte diffusion. The excellent electrocatalytic activity of the sensor was attributed to the uniform distribution of Fe^2+^ and Co^2+^ active centers within the MOF structure, which facilitates the electrooxidation of lactic acid to pyruvic acid. Moreover, the sensor exhibited a broad linear range from micromolar to high millimolar concentrations, a low micromolar detection limit, and good selectivity against common sweat interferents, demonstrating promising performance for wearable point-of-care lactic acid monitoring. In another study, a copper-nickel-BTC MOF was integrated with eco-friendly, biomass-derived chitosan-cationic guar gum hydrogels for the trace determination of acetaminophen [75]. Acetaminophen is a widely used analgesic/antipyretic whose accurate detection in biological and environmental samples is important for overdose diagnosis and environmental monitoring due to its prevalence and potential toxicity at high concentrations. This work combines natural polymer-based hydrogels with catalytically active bimetallic MOF nanoparticles to create a flexible, conductive sensing interface that enhances analyte diffusion and electron transfer. The MOF/hydrogel composite exhibits a three-dimensional network structure with a high specific surface area, improved conductivity, and strong interaction with acetaminophen molecules, enabling sensitive electrochemical oxidation responses. The sensor achieved a wide linear range down to trace concentrations with a low detection limit suitable for clinical and environmental analysis, and demonstrated good selectivity against common interferents, indicating robust practical applicability for rapid, portable acetaminophen monitoring (Figure 4). Overall, these studies demonstrate that bimetallic MOF-based electrochemical sensors, particularly when integrated with flexible hydrogel or wearable platforms, exhibit enhanced electrocatalytic activity, improved charge-transfer kinetics, and increased analyte accessibility arising from synergistic metal–metal interactions. The coexistence of two metal centers within the MOF framework promotes complementary redox activity, modulates the electronic structure, and generates a higher density of catalytically active sites compared to monometallic counterparts. When embedded within hydrogel matrices, the porous MOF structure facilitates efficient mass transport, while the hydrogel provides a three-dimensional architecture with high mechanical compliance, biocompatibility, and stable biointerfacing. Such a hybrid architecture enables sensitive, selective, and minimally invasive detection of diverse biochemical markers in complex biofluids, including tears, sweat, and other physiological media. Collectively, these findings highlight the strong potential of bimetallic MOF–hydrogel systems as advanced platforms for next-generation wearable and point-of-care electrochemical sensing applications.

A composite paper-based electrochemical aptasensor was developed by integrating a copper MOF within a chitosan/polyacrylamide hydrogel on a paper substrate for ultrasensitive detection of aflatoxin B1, which is a potent mycotoxin produced by *Aspergillus* species that poses serious health risks, including liver cancer and immunosuppression, making its low-level detection crucial for food safety monitoring [76]. The Cu–TCCP MOF possesses a high surface area and abundant active sites, enabling efficient immobilization of aflatoxin-specific DNA aptamers. The three-dimensional hydrogel network further facilitates higher MOF loading, enhances mass transport, and provides a stable and robust electrode interface. Upon binding of aflatoxin to the aptamer, steric hindrance and partial blockage of diffusion pathways occur, modulating ion transport at the electrode surface and generating a dual-amplified electrochemical signal. Similarly, a MIP-based electrochemical sensor was constructed using a Cu-BTC MOF integrated with sodium alginate-chitosan for highly selective detection of chelerythrine, which is a benzylisoquinoline alkaloid with significant pharmacological activity but also potential cytotoxic and inflammatory effects at elevated levels, making its accurate trace analysis important for herbal medicine quality control and food safety [77]. The work employed a molecular imprinting strategy on the Cu-BTC MOF to create specific recognition sites for chelerythrine, while the biopolymer enhanced film formation and mechanical stability on the electrode surface. The hierarchical porous structure and high surface area of the imprinted Cu-BTC MOF facilitated efficient analyte binding and electron transfer. Under optimized conditions, the sensor exhibited a wide linear response and low detection limit for chelerythrine, with excellent sensitivity against structurally related interferents and reliable performance in real sample matrices, demonstrating potential for practical electrochemical monitoring of trace alkaloids.

In another study, a 3D bio-printing vascular microtissue electrochemical biosensor was developed for parvalbumin detection by combining a graphite rod electrode modified with a Cu-BTC MOF and a conductive hydrogel containing polydopamine-functionalized multiwalled carbon nanotubes mixed with mast and endothelial cells [78]. Fish parvalbumin is a major allergenic protein responsible for immune reactions in susceptible individuals, and its rapid detection in food products is important for food safety and allergy prevention. The developed platform leverages the high surface area and intrinsic electrocatalytic properties of the Cu-BTC MOF to enhance current response, while the 3D-printed vascular microtissue provides a biomimetic sensing interface that mimics physiological allergen-receptor interaction. Moreover, the developed hydrogel showed no significant cytotoxic effects relative to the control group. Collectively, these findings highlight the strong potential of single-metal Cu-MOF systems for delivering high sensitivity, tunable framework architecture, and versatility across various electrochemical recognition strategies. To further broaden this perspective, the next two studies briefly explore Zn-MOF and Zr-MOF systems, where distinct metal coordination environments and superior chemical stability offer complementary advantages for electrochemical sensing applications. Two hydrogel-based electrochemical biosensors were developed by embedding zeolitic imidazolate framework-8 (ZIF-8) MOF nanoparticles within a 3D polymeric hydrogel network to achieve stable enzymatic [79] and non-enzymatic [80] glucose sensing. In the former work, the ZIF-8 MOF served as a protective encapsulation matrix for glucose oxidase, enhancing enzyme stability against temperature variations and preserving catalytic activity. The hydrogel network amplified the impedance response and supported stable signal generation across a broad temperature range. Electrochemical measurements showed a linear relationship between impedance and glucose concentration over a relevant range, with strong anti-interference capability and validated accuracy in honey samples, indicating the effectiveness of the MOF/hydrogel design for robust, enzyme-based electrochemical detection. While in the latter work, the sensor was fabricated by anchoring GNPs onto carbonized ZIF-8 and integrating them onto a flexible screen-printed carbon electrode modified with calcium alginate gel for on-skin sweat analysis. The carbonized ZIF-8 provides a high surface area conductive scaffold, while the anchored GNPs exhibit intrinsic oxidase-like and peroxidase-like catalytic activities, enabling cascade reactions that mimic enzymatic glucose oxidation. The soft calcium alginate layer enhances biocompatibility, sweat uptake, and mechanical conformity to skin surfaces, facilitating continuous electrochemical response. The sensor demonstrated a linear detection range covering typical sweat glucose concentrations with a low limit of detection, and its flexibility and antibacterial properties supported stable, real-time glucose monitoring during wear, showcasing promise for noninvasive diabetes tracking. Although both studies focus on glucose sensing using MOF-based hydrogels, they differ substantially in design philosophy and application domain. The former approach employs the MOF primarily to protect and stabilize the biological enzyme within the hydrogel matrix, thereby enhancing enzyme preservation and ensuring robust analytical performance. In contrast, the latter wearable platform eliminates the use of natural enzymes and instead utilizes a nanozyme-like, MOF-derived catalytic interface integrated within a soft hydrogel encapsulation. This strategy enables flexible, noninvasive sweat monitoring while balancing catalytic efficiency, mechanical compliance, user comfort, and antibacterial functionality.

**Figure 5:**
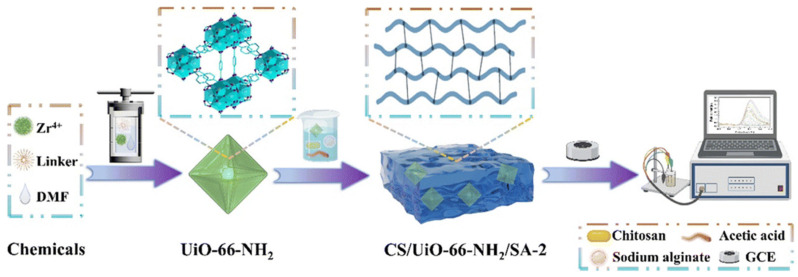
Scheme for the synthesis of CS/UiO-66-NH_2_/SA-2 sensor and its electrochemical activity. Reproduced with permission from [81].

Similarly, a 3D electroanalytical sensing platform was developed by embedding amino-functionalized zirconium-based MOF (UiO-66-NH_2_) within a chitosan/sodium alginate hydrogel network for sensitive electrochemical detection of chlorogenic acid in food samples [81]. The stepwise fabrication of the sensor is shown in Figure 5. Chlorogenic acid is an important polyphenolic antioxidant abundant in fruits and beverages, with relevance to nutritional quality and potential health benefits, making its accurate quantification in foods critically important. The hierarchical UiO-66-NH_2_ provided a high surface area and abundant active sites, while the hydrogel’s interconnected network facilitated efficient mass transport and analyte enrichment at the electrode interface. Electrochemical measurements demonstrated a wide linear response and a low detection limit, attributable to the synergistic combination of porous MOF structure and 3D hydrogel architecture. Excellent analytical performance with recoveries near 100% in real apple and coffee samples highlighted the sensor’s practical applicability for food analysis. Beyond pristine single-metal MOFs, recent studies have increasingly explored metal oxide-derived MOF architectures, in which controlled transformation or hybridization enhances electrical conductivity and catalytic activity. These modifications improve charge-transfer kinetics and active site accessibility, thereby significantly boosting electrochemical sensing performance. A conductive hydrogel sensor was developed by embedding a ZnS/MnO_2_ MOF into a polyvinyl alcohol/Nafion matrix for selective electrochemical detection of glutathione in serum samples [82]. Glutathione is a vital intracellular antioxidant involved in redox homeostasis, detoxification, and cellular protection, and its dysregulation is linked to oxidative stress, aging, and various pathological conditions, making sensitive detection in biological fluids clinically significant. The ZnS/MnO_2_ MOF-hydrogel composite exhibits a highly porous structure with abundant active sites, enhanced electrical conductivity, and oxygen/sulfur vacancies that facilitate efficient electron transfer and electro-oxidation of glutathione at the electrode interface. Morphological studies confirmed uniform dispersion of MOF particles throughout the hydrogel, supporting effective analyte access and signal transduction. The sensor showed a broad linear detection range, a low detection limit, and strong selectivity against common interferents, with high recovery in real serum samples, demonstrating its promise for sensitive physiological glutathione analysis.

In addition, a flexible, self-standing 3D hydrogel-derived foam electrode was engineered by incorporating Nb_2_O_5_/Al-BDC MOF nanocomposite into a porous hydrogel foam for the electrochemical detection of the antituberculosis drug delamanid in biological fluids [83]. Delamanid is a critical therapeutic for drug-resistant tuberculosis, and its sensitive monitoring is important for optimized dosing and avoiding toxicity. The Nb_2_O_5_/Al-BDC MOF composite combined redox-active Nb species with the high surface area and porosity of the MOF, while the hydrogel-derived foam provided an interconnected, mechanically flexible matrix that facilitated electrolyte diffusion and electron transport. Structural analysis confirmed successful anchoring of Nb_2_O_5_ on the Al-BDC MOF framework, enhancing electrocatalytic activity toward delamanid oxidation. Electrochemical sensing exhibited a wide linear range from nanomolar to micromolar concentrations with a low detection limit and good selectivity against interferents, underscoring its potential for sensitive, flexible biosensing applications.

Beyond metal oxide-derived MOF architectures, recent studies have further advanced toward metal nanoparticle-decorated MOF systems, where nanoscale metallic sites introduce enzyme-mimetic activity and enhanced electrocatalytic efficiency for wearable and noninvasive sensing. Recently, a self-supported PtCu hydrogel-based MOF sensor was constructed by in situ growth of a Cu-BTC MOF onto a conductive Pt-Cu hydrogel scaffold for sensitive electrochemical detection of o-sec-butylphenol in water samples [84]. This o-sec-butylphenol is a hazardous organic pollutant commonly found in industrial effluents and environmental waters, posing risks to ecological systems and human health, which necessitates rapid and accurate analytical methods for trace monitoring. The coordinated PtCu hydrogel network served as a robust support that enhanced electrical conductivity, structural stability, and active site exposure, while the Cu-BTC MOF layer provided a large specific surface area and strong adsorption affinity toward phenolic pollutants. The resulting sensor exhibited a wide linear response to o-sec-butylphenol concentrations from low micromolar to higher levels, along with good selectivity and repeatability in water samples, highlighting its potential for practical environmental pollutant sensing.

In addition to metals, metal oxides, and metal nanoparticles-based MOF hybrid systems, polymer-based MOF composites have emerged as versatile platforms in electrochemical sensing due to their tunable physicochemical properties and facile functionalization. Recently, a dual-responsive electrochemical sensing platform was constructed using a polyacrylamide/Fe-MOF/zinc-finger-peptide hydrogel as a silent layer combined with a sodium alginate-Ni^2+^-graphene oxide hydrogel as the signal layer for ultrasensitive detection of pro-gastrin-releasing peptide [85]. Pro-gastrin-releasing peptide is an important tumor biomarker associated with small-cell lung cancer, and its accurate detection is critical for early diagnosis and disease monitoring. The sensing mechanism relies on GOx@ZIF-8 immunoprobes that catalyze glucose to generate hydrogen peroxide and gluconic acid, triggering ZIF-8 decomposition and Zn^2+^ release. The released Zn^2+^ specifically coordinates with cysteine and histidine residues in the zinc-finger peptide, while Fe-MOF undergoes Fe^3+^/Fe^2+^ conversion to generate free radicals that disrupt the hydrogel structure, leading to rapid impedance changes. The platform exhibited fast response, high sensitivity, low detection limits, and good selectivity, demonstrating an efficient polymer-Fe-MOF hybrid strategy for responsive electrochemical biosensing. Another polymer-based MOF work was reported, where a biomimetic electrochemical sensor was engineered by integrating intestinal villi microtissue with a composite of Cu-BTC MOF modified with polydopamine and phenylboronic acid MIPs for selective detection of gelatin protein [86]. Gelatin is a major protein component in food products and biological systems, and its accurate quantification is important for nutritional labeling, quality control, and detecting adulteration in complex matrices. The work leverages the high surface area and affinity of Cu-BTC MOF, combined with the specificity of phenylboronic acid functionalized MIPs and the biomimetic microtissue interface, to enhance selective binding and electron transfer kinetics. Structural characterization confirmed effective imprinting and biomimetic integration, while electrochemical investigation exhibited precise quantification of gelatin allergens in fish meat. Overall, polymer-integrated MOF systems represent a promising strategy for engineering multifunctional and stimulus-responsive electrochemical sensors, paving the way for next-generation biosensing platforms with enhanced specificity and adaptability. The electroanalytical performances of various MOF-integrated hydrogel-based sensors are compared in Table 1.

## 7. MXene-Integrated Hydrogel for Electrochemical Health Monitoring

A portable bacterial strip sensor was developed for the selective detection of *Porphyromonas gingivalis* (*Pg*) using a multifunctional hydrogel incorporating GNPs decorated vanadium carbide MXene (V_4_C_3_-MX) nanosheets and ferrocene carboxaldehyde [87]. Pg is a key periodontal pathogen and has been implicated in several systemic diseases, including Alzheimer’s disease, cardiovascular disorders, and cancer [88]. The developed hydrogel-based sensor exhibited excellent adhesiveness, enhanced electrical conductivity, and improved operational stability. Notably, it enabled highly sensitive and accurate quantification of *Pg* in clinical saliva samples. Similarly, a disposable strip-type microRNA sensor was developed using Ti_3_C_2_ MX aerogel integrated with GNPs for the sensitive detection of miRNA-155 [89]. miRNA-155 is one of the highly studied microRNAs, playing a critical role in regulating immune responses and inflammation; its dysregulation has been associated with various pathologies, including cancer and autoimmune disorders. The sensing matrix was constructed from a composite of Ti_3_C_2_T_x_ MX, graphene oxide and ethylenediamine, followed by electrodeposition of GNPs to form the desired sensor interface. Notably, the sensor enabled reliable quantification of miRNA-155 in clinical samples, demonstrating its capability for effective detection of circulating microRNAs in complex biological matrices. In another study, a nanoengineered hydrogel-based sensor was developed by integrating Ti_3_C_2_T_x_ MX into a conductive hydrogel matrix, further decorated with GNPs and a ferrocene-tagged aptamer for the selective detection of estradiol [90]. Estradiol, a hormone primarily produced in the ovaries, plays a vital role in uterine and ovarian development, as well as in cardiovascular function and bone health. The hydrogel network was fabricated by crosslinking carboxymethyl chitosan with sodium carboxymethyl cellulose, providing excellent antifouling characteristics and improved biointerface stability. The synergistic incorporation of MX and GNPs markedly enhanced electrical conductivity and facilitated efficient electron transfer, thereby improving electrocatalytic performance. As a result, the sensor exhibited reliable operation in complex clinical samples, achieving high sensitivity, selectivity, and analytical accuracy.

**Figure 6:**
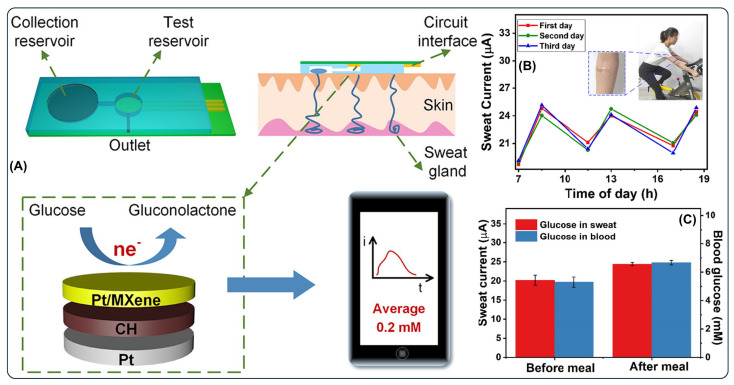
(**A**) Graphical illustration of a microfluidic channel and Pt/MX hydrogel sensor on the skin surface with a display module. (**B**) Real-time sweat glucose monitoring using the developed sensor during exercise. (**C**) Current changes measured by the sweat glucose sensor before and after a meal in comparison with blood glucose. Reproduced with permission from [91].

**Figure 7:**
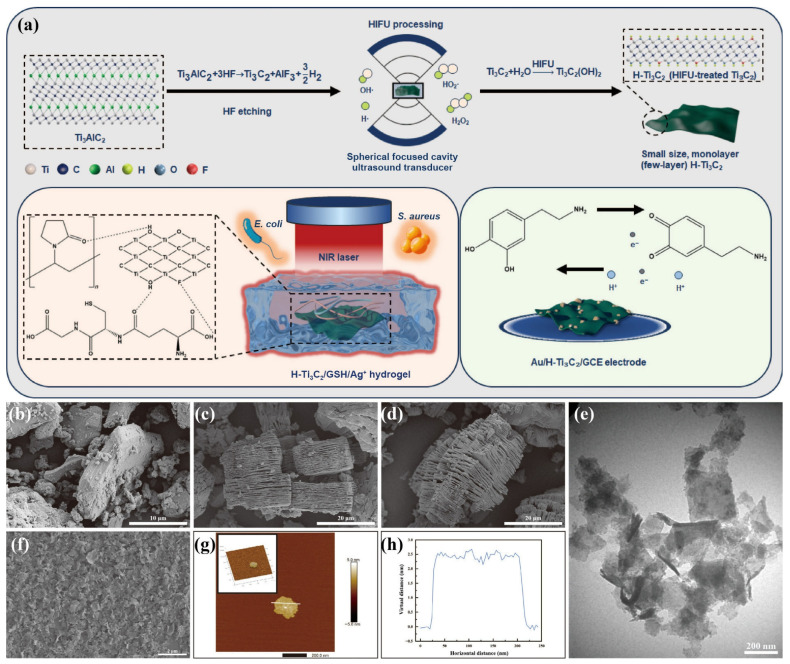
(**a**) Scheme for the synthesis of H-Ti_3_C_2_ nanosheets and their photothermal and electrochemical applications. SEM images of (**b**) Ti_3_AlC_2_, (**c**,**d**) Ti_3_C_2_. (**e**) TEM, (**f**) SEM, (**g**) AFM images, and (**h**) cross-sectional analysis of H-Ti_3_C_2_. Reproduced with permission from [92].

A wearable non-enzymatic sweat glucose sensor was developed using a conductive hydrogel enclosed with platinum nanoparticles (PtNPs)-decorated Ti_3_C_2_T_x_ MX [91]. The work synthesizes Pt/MX hybrid catalysts, leveraging the high electrical conductivity and hydrophilicity of MXs to support uniform Pt distribution and facilitate charge transport. Immobilization of this hybrid with hydrogel enhanced mechanical flexibility and maintained conformal skin contact during wear. The sensor was integrated into a microfluidic system, which collects the sweat and transports it to the sensor surface, by which the sweat glucose was quantified through a smartphone display, as shown in Figure 6. Similarly, a Ti_3_C_2_ MX-incorporated hydrogel-based sensor was developed for dopamine detection [92]. Dopamine is a critical neurotransmitter involved in central nervous system signaling, and its dysregulation is linked to neurological disorders such as Parkinson’s and schizophrenia, making sensitive and selective detection important for clinical diagnostics. In this study, structurally regulated monolayers of Ti_3_C_2_ MX nanosheets were prepared using a high-intensity focused ultrasound method, producing MX sheets with enhanced photothermal and electrochemical properties. The synthesis strategy and morphological characterization of the fabricated hydrogel-based sensor are illustrated in Figure 7. The hydrogel matrix facilitated efficient electrolyte diffusion and improved analyte accessibility, while the incorporated MX provided rapid electron transport pathways and abundant active sites for dopamine oxidation. Further incorporation of GNPs into the MX–hydrogel composite led to enhanced electrocatalytic currents, which were attributed to the synergistic interaction between GNPs and MX within the conductive hydrogel network. Overall, these studies demonstrate that the hydrogel matrix plays a crucial role in enhancing operational stability, interfacial adhesion, and antifouling performance of the sensor, while simultaneously providing a hydrated and flexible platform for efficient analyte diffusion. Concurrently, the decoration of MX nanosheets with noble metal nanoparticles significantly improves electrical conductivity and electrocatalytic activity, owing to synergistic enhancements in charge-transfer kinetics and active site density within the composite architecture.

Another wearable electrochemical sweat sensor for dopamine was designed using an antifouling and antimicrobial composite hydrogel incorporating Ti_3_C_2_ MX and cerium oxide nanorods [93]. The sensing interface was fabricated by integrating MX nanosheets and CeO_2_ nanorods into a hydrophilic protein-based hydrogel matrix, exploiting the high electrical conductivity and electrocatalytic activity of MX to enhance charge-transfer efficiency and signal output. Meanwhile, CeO_2_ nanorods contributed additional catalytic activity and intrinsic antimicrobial properties, effectively mitigating biofouling from skin flora and sweat constituents. The hydrophilic polymer network formed a stable hydration layer that reduced nonspecific adsorption and facilitated consistent analyte diffusion to the active sites. As a result, the sensor achieved sensitive and selective dopamine detection in sweat, demonstrated good operational stability under repeated use, and exhibited effective antibiofouling performance, highlighting its potential for long-term wearable monitoring applications. A composite hydrogel sensor was formulated by integrating titanium oxide/Ti_3_C_2_ MX nanostructures with a polyvinyl alcohol/graphene oxide hydrogel and applied as a modifying layer on a screen-printed carbon electrode for urinary norepinephrine detection [94]. Norepinephrine is a key catecholamine neurotransmitter involved in autonomic nervous system regulation and is a clinical marker for neurological disorders, stress response, and cardiovascular conditions, making its sensitive monitoring in biological fluids important for early diagnosis and screening. In this design, the TiO_2_/MX provided high electrical conductivity, abundant electroactive sites, and enhanced electrocatalytic activity toward norepinephrine oxidation, while the PVA/GO hydrogel network facilitated auto-sample preconcentration, efficient mass transport, and mechanical stability of the composite interface. Collectively, both sensors leverage metal oxide-MX synergism within hydrogel matrices; however, their functional priorities differ. The TiO_2_/MX system primarily focuses on enhancing catalytic oxidation efficiency and analytical sensitivity for detecting a clinical catecholamine in biofluids. In contrast, the CeO_2_/MX composite emphasizes antifouling stability in addition to catalytic performance, making it particularly suitable for continuous operation in wearable sweat-monitoring environments. These contrasting design strategies demonstrate how the choice of metal oxide can be rationally tailored to align with specific analyte chemistry, sensing mechanisms, and operational conditions in MXene-based hydrogel sensors.

**Figure 8:**
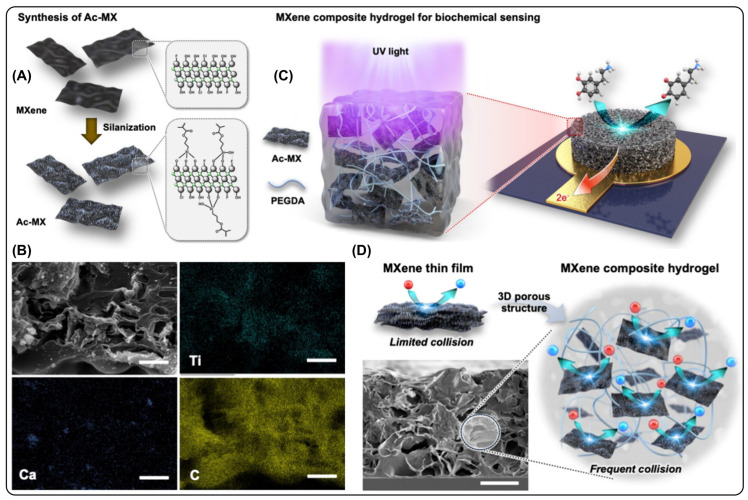
(**A**–**C**) Scheme for the synthesis of Ac-MX-based hydrogel and its electrocatalytic activity. (**B**) SEM-EDX mapping of the hydrogel. (**D**) Graphical visualization of the 3D porous structure of the hydrogel in comparison with the MXene thin film. Reproduced with permission from [95].

A silane-functionalized Ti_3_C_2_T_x_ MX was chemically incorporated into a poly(ethylene glycol) diacrylate hydrogel network to fabricate a conductive composite for enhanced electrochemical sensing of neurotransmitters, including dopamine and serotonin, as well as antioxidants such as uric acid in biological fluids [95]. Along with dopamine, serotonin also plays a crucial role in neural signaling, and its precise quantification is important for diagnosing and monitoring neurological disorders as well as assessing oxidative stress via antioxidant levels. In this design, silane functionalization improved the interfacial compatibility between MX nanosheets and the hydrogel, promoting uniform dispersion and strong polymer–MX coupling. This interfacial engineering enhanced electron transport efficiency and reinforced structural integrity (Figure 8). Meanwhile, the hydrated hydrogel network provided a flexible and biocompatible environment that facilitated ion diffusion and improved analyte accessibility to the electroactive MXene surfaces.

A wearable electrochemical sweat glucose sensor was developed based on a one-step synthesized Ti_3_C_2_T_x_MX-functionalized poly(3,4-ethylenedioxythiophene)-poly(styrene sulfonate), PEDOT:PSS conductive polymer hydrogel, designed for noninvasive and continuous monitoring of glucose [96]. In this work, Ti_3_C_2_T_x_ MX nanosheets were integrated into a PEDOT:PSS hydrogel matrix, where MX provided high electrical conductivity, porosity, and mechanical flexibility, and the PEDOT:PSS network supported ionic transport and conformal contact with skin. The resulting composite hydrogel exhibited robust mechanical properties and a porous network favorable for sweat uptake and analyte diffusion. Similarly, a continuous glucose monitoring system was developed by applying a protein-based hydrogel coating with enhanced anti-biofouling properties onto the sensors to achieve long-term, accurate, and point-of-care monitoring [97]. The protein-based hydrogel coating was specifically designed to resist non-specific adsorption of proteins and cellular debris, which often compromise sensor performance during prolonged operation in physiological environments. The hydrated hydrogel network established a biocompatible interface that preserved analyte accessibility while mitigating surface biofouling. Additionally, the incorporation of Ti_3_C_2_T_x_ MX-polydopamine composites within the hydrogel enhanced electrical conductivity and improved the chemical stability of the coating. The developed hydrogel demonstrated excellent cell viability toward L929 cells and did not exhibit significant cytotoxic effects. When applied to electrochemical glucose sensors, the anti-biofouling hydrogel resulted in more stable baseline currents, reduced signal drift during extended operation, and improved reproducibility in point-of-care settings.

In addition, a wearable, self-powered electrochemical sensor was constructed by integrating Ti_3_C_2_T_x_ MX nanosheets into a polypyrrole/polyurethane conductive hydrogel composite and coupling it with enzyme-modified electrodes for noninvasive lactate detection during physical activity [98]. Lactate is a key metabolite reflecting anaerobic metabolism and exercise intensity, and its real-time monitoring in sweat enables performance tracking and early detection of metabolic stress. The composite hydrogel served as a self-powered sensing interface paired with activated carbon cloth electrodes functionalized with lactate oxidase and bilirubin oxidase on reduced graphene oxide supports, forming a biofuel cell architecture. Integration of the MX hydrogel boosted current output nearly 15-fold by improving electron mobility and ionic conduction within the matrix. In another study, a highly sensitive electrochemical immunosensor was developed by integrating a double-conductive antifouling hydrogel matrix with Ti_3_C_2_T_x_MX and conductive PEDOT:PSS for ultrasensitive detection of carcinoembryonic antigen in complex serum samples [99]. Carcinoembryonic antigen is a clinically significant cancer biomarker whose trace analysis in serum is important for early diagnosis, prognosis, and monitoring of treatment responses. MXene nanosheets functioned as the primary conductive framework, offering high electrical conductivity and intrinsic hydrophilicity that contributed to antifouling performance by reducing nonspecific adsorption of serum proteins. The incorporation of PEDOT:PSS further improved electrical conductivity and reinforced structural stability within the composite matrix. Additionally, the three-dimensional hydrogel network encapsulated an internal redox standard, hexaammine ruthenium (III) ions, enabling signal normalization and minimizing variability arising from environmental fluctuations or instrumental drift. A flexible, Ti_3_C_2_T_x_MX-decorated polymer dot hydrogel sensor was engineered to respond to cancer cell activity by tuning its elastic modulus, porosity, and electrical conductivity for potential biomedical sensing [100]. Cancer cell metabolites and microenvironment changes are important indicators for early tumor detection and therapy monitoring, and materials that can respond mechanically and electrically to these biological cues offer promising routes for responsive sensing and diagnostics. Here, 2D MX nanosheets were integrated with polymer dots dispersed within a flexible hydrogel, resulting in a composite network whose mechanical and conductive properties could be modulated in the presence of cancer cell secretions.

A printable conductive hydrogel platform for dopamine monitoring was developed by combining a biopolymer alginate matrix with 2D Ti_3_C_2_T_x_MX nanosheets to create self-supporting electroactive structures through extrusion-based 3D printing [101]. In this work, alginate functioned as the primary polymer scaffold, providing mechanical integrity, ionic conductivity, and shape fidelity after crosslinking, while MX nanosheets established continuous electronic conduction pathways within the hydrogel network. The resulting polymer–MX composite enabled the fabrication of flexible, free-standing hydrogel electrodes without reliance on rigid substrates. Beyond sensing, the hydrogel’s electroactivity enabled electrically triggered drug release, highlighting the multifunctional capability of polymer–MX hydrogels as integrated sensing and actuation interfaces for soft bioelectronic and biomedical applications. Collectively, polymer–MX conductive hydrogels exemplify how deliberate polymer architecture combined with the exceptional conductivity and surface functionality of MX can produce robust electrochemical sensing interfaces. The synergistic integration enhances mechanical adaptability, suppresses biofouling, and ensures reliable signal transduction under physiologically relevant conditions. Such hybrid systems demonstrate remarkable versatility, effectively addressing a broad spectrum of analytical challenges, including metabolite and neurotransmitter monitoring, immunosensing, and the development of integrated or self-powered sensing platforms.

Beyond these well-defined material categories, a label-free electrochemical immunosensor was developed by coating a screen-printed electrode with an electroactive, anti-biofouling hydrogel composite containing a crosslinked protein network integrated with Ti_3_C_2_T_x_ MX nanosheets for sensitive detection of the chemokine IP-10 [102]. This chemokine IP-10 is an inflammatory biomarker associated with acute kidney transplant rejection, and its accurate quantification in serum can enable early diagnosis and personalized post-transplant monitoring without invasive biopsies or lengthy assays. In this design, a three-dimensional bovine serum albumin hydrogel was crosslinked with glutaraldehyde to form a porous polymer network, into which MX was incorporated to impart electrocatalytic activity and enhance charge-transfer efficiency. The hydrogel exhibited antifouling properties that minimized nonspecific protein adsorption from serum, thereby stabilizing the immunosensor interface and preserving analytical performance. The electroanalytical performances of various MXene-integrated hydrogel-based sensors are compared in Table 2.

## 8. MXene-MOF-Integrated Hydrogel for Electrochemical Health Monitoring

The integration of MXs and MOFs within conductive hydrogel matrices represents an emerging strategy that aims to synergistically combine high electrical conductivity with molecular selectivity and catalytic activity. Although this hybrid material concept is still in its initial stages, it demonstrates its potential to overcome the individual limitations of MX- and MOF-based hydrogels by enabling efficient charge transport alongside enhanced analyte interaction. The following paper discusses the first reported electrochemical sensing platform employing an MX-MOF hybrid hydrogel, highlighting its material design and sensing performance.

A flexible electrochemical sensing platform was developed using a cobalt-based MOF and vanadium carbide (V_2_C) MX hybrid embedded within a 3D hydrogel matrix for simultaneous detection of levothyroxine and carbamazepine in simulated blood serum [103]. Both levothyroxine and carbamazepine are clinically relevant pharmaceuticals whose levels in biological fluids are important for thyroid disorder management and anticonvulsant therapy monitoring, respectively. The MOF provided a high surface area and abundant coordination sites for analyte interaction, while layered MX nanosheets enhanced electrical conductivity and facilitated rapid electron transfer. The hydrogel served as a flexible, porous network that supported ion mobility and improved interface contact between the hybrid and the electrolyte. Structural analysis confirmed an interconnected porous architecture integrating MOF and MX components. Further, EIS analysis demonstrated a ~12-fold reduction in charge-transfer resistance for the MX-MOF-hydrogel composite relative to the bare hydrogel, indicating significantly enhanced interfacial electron-transfer kinetics. However, detailed physicochemical investigations of electrical double-layer modulation in such ternary systems remain limited and warrant further study. Furthermore, the electrocatalytic investigation showed wide linear detection ranges with low nanomolar limits of detection and good selectivity against common interferents in complex matrices, underscoring its promise for biomedical sensing. Although MOF-MX hybrid hydrogel exhibits promising electrochemical performance, the interfacial charge-transfer mechanisms between semiconducting MOFs and metallic MX remain largely unexplored. Differences in electronic structure and work function may promote interfacial charge redistribution and facilitate electron transport, with MXs serving as conductive pathways and MOFs providing catalytic or selective sites. However, systematic studies on band alignment and interface energetics are still lacking, highlighting an important direction for future investigation.

## 9. Summary, Challenges and Future Outlook

Smart health monitoring has emerged as a cornerstone of next-generation healthcare, driven by the need for continuous, noninvasive and personalized physiological assessment. In this context, conductive hydrogel-based electrochemical sensors have gained significant attention as soft, tissue-compatible interfaces capable of overcoming the mechanical mismatch and signal instability associated with traditional rigid electrodes. Their intrinsic flexibility, high water content and interconnected polymeric networks enable intimate contact with biological surfaces, supporting reliable signal transduction under dynamic conditions. The incorporation of functional nanomaterials, including MXs and MOFs, has further expanded the capabilities of hydrogels by enhancing electrical conductivity, catalytic activity, porosity, and analyte selectivity. These hybrid-hydrogel architectures demonstrate improved electrochemical performance across a broad range of health-monitoring applications, from metabolite and biomarker detection to wearable and implantable sensing platforms.

Although MX- and MOF-integrated hydrogels have emerged as promising building blocks for advanced electrochemical health-monitoring platforms, they still exhibit intrinsic limitations that must be addressed to ensure reliable long-term operation. MXs are susceptible to oxidation under ambient and physiological conditions, which can progressively degrade their electrical conductivity and electrochemical stability. To mitigate this issue, various strategies have been explored, including surface passivation, incorporation of antioxidants, heterostructure formation, and encapsulation within protective matrices to improve oxidation resistance and durability. In contrast, many MOF structures suffer from limited intrinsic electrical conductivity and potential structural instability in aqueous or biological environments. These drawbacks can be alleviated through bimetallic or multimetallic engineering, conversion to MOF-derived conductive composites, hybridization with conductive nanomaterials and polymer reinforcement to enhance both charge-transfer efficiency and mechanical robustness. While the incorporation of MXs and MOFs improves the electrical conductivity of hydrogels, challenges such as mechanical fatigue under repeated deformation and swelling-induced structural changes can still compromise signal stability. These limitations can be addressed by designing double-network or self-healing hydrogel architectures, optimizing crosslinking density, and incorporating antifouling or antibacterial components to improve durability and interfacial stability [39]. Compared with conventional commercial electrochemical platforms, MX- and MOF-integrated hydrogel sensors often demonstrate lower limits of detection and higher analytical sensitivity under laboratory conditions due to enhanced catalytic interfaces and improved ion-electron coupling. However, commercial systems currently outperform academic prototypes in terms of long-term operational stability, standardized calibration, regulatory validation, and scalable manufacturing. The competitive advantage of hydrogel-based systems lies primarily in their mechanical compliance, conformal biointerfacing, and potential integration into next-generation wearable and closed-loop architecture rather than immediate replacement of established clinical devices. Overall, the rational integration of MX and MOFs within engineered hydrogel matrices enables synergistic enhancements in charge-transfer kinetics, catalytic activity, interfacial stability, and mechanical compliance, thereby advancing the development of robust, sensitive, and wearable electrochemical health-monitoring systems.

Looking forward, the evolution of conductive hydrogel-based electrochemical sensors is expected to progress toward multifunctional hydrogel systems that seamlessly integrate sensing, actuation, and therapeutic functionalities within a single soft interface. Rather than serving solely as passive sensing layers, future hydrogels are likely to function as active bioelectronic platforms capable of simultaneously monitoring physiological signals and delivering targeted interventions. The intrinsic softness and conformability of hydrogels make them particularly attractive for long-term integration with biological tissues, enabling stable operation in wearable, epidermal, and implantable devices. Beyond biochemical monitoring, conductive hydrogels are increasingly being explored for electrophysiological signal acquisition, including electrocardiography (ECG), electroencephalogram (EEG), electromyography (EMG), and electrooculogram (EOG) [104,105]. Their ability to maintain intimate contact with skin and soft tissues reduces motion artifacts and improves signal fidelity compared to rigid electrodes. Moreover, hydrogel-based electrodes can support electrical stimulation for neuromodulation, muscle rehabilitation, tissue regeneration and pain management, as well as controlled drug delivery, where electrical cues trigger on-demand release of therapeutic agents [106,107,108]. Such multifunctional capabilities position conductive hydrogels as promising platforms for simultaneous monitoring and therapy, enabling responsive and adaptive healthcare solutions.

**Figure 9:**
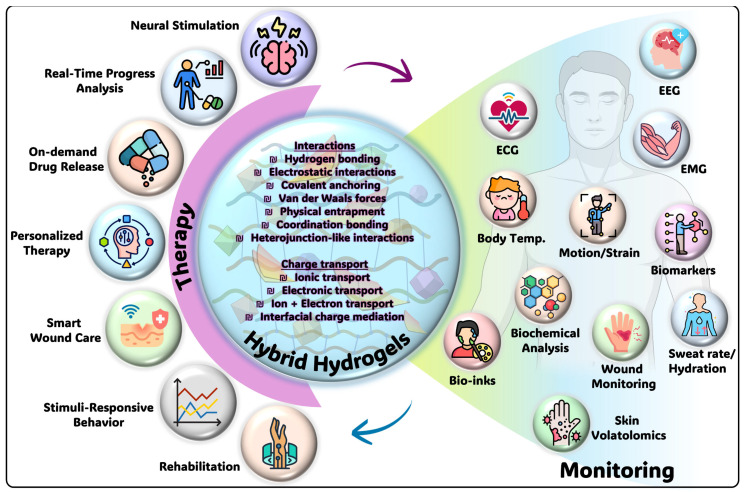
Conceptual illustration of next-generation hybrid conductive hydrogel systems for closed-loop healthcare applications. The icons used in this illustration were adapted from www.flaticon.com.

Beyond materials engineering, recent advances in data science have begun to reshape how electrochemical sensor outputs are interpreted and utilized. Conductive hydrogel-based sensors, particularly in wearable formats, generate continuous and high-dimensional electrochemical data streams that are well-suited for artificial intelligence (AI) and machine learning (ML) analysis. Data-driven models can enhance signal interpretation by compensating for noise, baseline drift, and inter-individual variability, which are common challenges in biological sensing. ML approaches may also enable pattern recognition and multi-analyte discrimination from complex electrochemical signatures, thereby expanding the analytical capabilities of hydrogel-based sensors [109,110]. However, despite the promise of AI and ML in electrochemical data processing, their integration with conductive hydrogel-based electrochemical sensors remains largely unexplored. To date, no systematic studies have evaluated specific model architectures or benchmarked algorithm performance for soft hydrogel platforms. Future research should therefore focus on coupling advanced data-driven approaches with hydrogel-based sensing systems to enhance signal reliability, long-term stability, and intelligent health-monitoring capabilities. Collectively, the integration of conductive hydrogel electrochemical sensors with AI-driven analytics may pave the way toward closed-loop health-monitoring systems (Figure 9). In such systems, real-time sensor data could be continuously analyzed to enable automated feedback mechanisms, including adaptive signal calibration, personalized diagnostics, or therapeutic interventions such as controlled drug delivery or electrical stimulation. The soft mechanics, biocompatibility, and long-term interface stability of hydrogel-based sensors make them particularly suitable for these closed-loop architectures, which require sustained and reliable interaction with biological tissues. As materials engineering, device integration, and intelligent analytics continue to advance, conductive hydrogel-based platforms are expected to play a central role in the development of autonomous, personalized, and next-generation smart healthcare technologies.

## Figures and Tables

**Table 1:** Electroanalytical performances of MOF-integrated hydrogels.

Sensor	Analyte	Techniques	Linear Range (µM)	LOD (µM)	Stability	Cost Analysis	Real Sample	Ref.
Ni-Co-BDC-PVA	Urea	DPV	5 × 10^−4^–0.1	781	-	$	Simulated tears	[72]
Ni-Co-BDC-CS-PAM	Adrenaline	DPV	0.5–2500	0.167	60 Cycles	$	Serum, Urine Milk	[73]
Fe-Co-BDC-PVA-CS	Lactic acid	SWV	0.05–0.1	0.01	4000 s	$	Simulated sweat	[74]
Cu-Ni-BTC-CHG	Acetaminophen	DPV	0.07–1500	0.023	30 Days, 60 Cycles	$	River & lake water, Cold remedy, Granules, Serum	[75]
Cu-TCCP-Apt-CS-PAM	Aflatoxin B1	DPV	1 × 10^−6^–0.1 *	3 × 10^−7^ *	10 Days	$ $ $	Peanuts	[76]
Cu-BTC-MIP-SA-CS	Chelerythrine	DPV	0.05–0.5	0.013	14 Days	$ $	Serum	[77]
Cu-BTC-PDA-MWCNT-GMA	Parvalbumin	DPV	0.1–2.5 *	0.065 *	12 h	$ $	Fish muscle	[78]
ZIF-8-GOx-CaCO_3_	Glucose	EIS	750–4000 *	-	60 °C, 2 h	$ $	Honey	[79]
ZIF-8-C-GNPs-CAG	Glucose	CA	10–300	4.99	-	$ $ $	Sweat	[80]
UiO-66-NH_2_-CS-SA-2	Chlorogenic acid	DPV	0.1–1000	0.03	30 Days, 50 Cycles	$ $	Apple, Coffee	[81]
ZnS-MnO_2_-MOF	Glutathione	DPV	0.01–10000	6.88 × 10^−3^	14 Days	$ $	Serum	[82]
Nb_2_O_5_-Al-BDC-HFF	Delamanid	DPV	0.001–20	8.3 × 10^−4^	14 Days	$ $ $	Urine, Serum	[83]
Cu-BTC-PtCu	o-sec-butylphenol	DPV	3–100	0.68	5 Cycles	$ $ $	Sewage water	[84]
Fe-MOF-PAM-ZIF-8	Pro-gastrin peptide	SWV	1 × 10^−7^–0.1 *	1.42 × 10^−8^ *	28 Days	$ $	-	[85]
Cu-BTC-PDA-PBA-MIP	Gelatin protein	DPV	0.01–10 *	2.8 × 10^−3^ *	-	$ $	Fish skin	[86]

Apt—aptamer; BDC—1,4-benzenedicarboxylate; BTC—1,3,5-benzenetricarboxylic acid; C—calcined; CA—chronoamperometry; CAG—calcium gels; CHG—cationic guar gum; CS—chitosan; DPV—differential pulse voltammetry; EIS—electrochemical impedance spectroscopy; GMA—gelatin methacryloyl; GOx—glucose oxidase; HFF—hydrogel derived foam; GNP—gold nanoparticles; MIP—molecularly imprinted polymer; MWCNT—multiwalled carbon nanotubes; PBA—3,5-difluorophenylboronic acid; PAM—polyacrylamine; PDA—polydopamine; PVA—polyvinyl alcohol; SA—sodium alginate; SWV—square wave voltammetry; TCCP—tetrakis(4-carboxyphenyl)porphyrin; ZIF—zeolitic imidazolate framework; *—µg mL^−1^; Comparative cost analysis: $ (low cost), $ $ (moderate cost), $ $ $ (high cost).

**Table 2:** Electroanalytical performances of MX-integrated hydrogels.

Sensor	Analyte	Techniques	Linear Range (µM)	LOD (µM)	Stability	Cost Analysis	Real Samples	Ref.
Gold-V_4_C_3_-MX	*Porphyromonas* *gingivalis*	DPV	10–1 × 10^7 #^	6 ^#^	14 Days	$ $ $	Saliva	[87]
Ti_3_C_2_T_x_MX-GNPs-CFP	miRNA-155	DPV	2 × 10^−7^–0.4	1.36 × 10^−14^	-	$ $ $	Clinical samples	[89]
GNPs- Ti_3_C_2_T_x_ MX -SPCE Apt	Estradiol	DPV	1 × 10^−7^–1 × 10^−3^ *	1.27 × 10^−7^ *	30 Days	$ $ $	Serum	[90]
Ti_3_C_2_T_x_ MX-PtNPs	Glucose	CA & LSV	0–1000	29.15	11 Days	$ $ $	Sweat	[91]
Gold- Ti_3_C_2_MX-GCE	Dopamine	AMP	1–1000	0.15	14 Days	$ $ $	-	[92]
CeO_2_-Ti_3_C_2_MX-AAH	Dopamine	AMP	0.05–300	0.017	25 Days, 50 Cycles	$ $	Sweat	[93]
TiO_2_- Ti_3_C_2_MX-PVA-GO	Norepinephrine	AMP	0.01–60	8000	14 Days	$ $	Urine	[94]
Ti_3_C_2_T_x_MX-PEGDA-CaCl_2_	Dopamine	DPV	2.5–200	2.55	40 Days	$ $	Serum	[95]
	Uric acid		10–100	25.11				
	Serotonin		1–100	0.83				
Ti_3_C_2_T_x_MX-PEDOT: PSS	Glucose	AMP	3–1294	1.9	10 Days	$ $	Sweat	[96]
Ti_3_C_2_T_x_MX-PDA-BSA	Glucose	CA	1 × 10^3^–1.8 × 10^4^	-	30 Days	$ $	-	[97]
Ti_3_C_2_T_x_MX-Ppy-PU-LOx-BOx-rGO-ACC	Lactate	CA	1–1 × 10^5^	-	30 Days	$ $ $	Sweat	[98]
CEA-Fc-dsDNA-K- Ti_3_C_2_T_x_MX-PEDOT:PSS-PNIPAM	CEA	DPV	1 × 10^−6^–1 *	4.1 × 10^−7^ *	15 Days	$ $ $	Serum	[99]
Ti_3_C_2_T_x_MX-PDA-HA dots	CHO-K1	EIS	-	2.58 ^†^	-	$ $	-	[100]
	B16F10			0.96 ^†^				
	MDA-MB-231			1.20 ^†^				
Ti_3_C_2_T_x_MX-Alginate	Dopamine	CV	0–1000	17	50 Cycles	$	-	[101]
Ti_3_C_2_T_x_MX-BSA-GA	Chemokine IP-10	CV	1 × 10^−6^–2 × 10^−8^ *	3.3 × 10^−6^ *	7 days	$	Serum	[102]

AAH—amyloid albumin hydrogels; ACC—activated carbon cloth; AMP—amperometry; Apt—aptamer; B16F10—mouse skin melanoma cells; BOx—bilirubin oxidase; BSA—bovine serum albumin; CA—chronoamperometry; CFP—carbon fiber paper; CEA—carcinoembryonic antigen; CHO-K1—Chinese hamster K1; CV—cyclic voltammetry; DPV—differential pulse voltammetry; EIS—electrochemical impedance spectroscopy; Fc-ds DNA—ferrocene-labeled double-stranded DNA; GCE—glassy carbon electrode; GA—glutaraldehyde; GNP—gold nanoparticles; GO—graphene oxide; MDA-MB-231—human breast cancer cell line; HA—hyaluronic acid; LOx—lactate oxidase; LSV—linear sweep voltammetry; NE—norepinephrine; PtNPs—platinum nanoparticles; PDA—polydopamine; PVA—polyvinyl alcohol; PEGDA—poly(ethylene glycol) diacrylate; PEDOT:PSS—poly (3,4-ethylenedioxythiophene):poly (styrene sulfonate); PNIPAM—poly (*N*-isopropylacrylamide); Ppy—polypyrrole; PU—polyurethane; rGO—reduced graphene oxide; SPCE—screen printed carbon electrode. *—µg mL^−1^; ^#^—CFU mL^−1^; ^†^—cells per well; Comparative cost analysis: $ (low cost), $ $ (moderate cost), $ $ $ (high cost).

## Data Availability

No data were used for the research described in the article.
